# Manipulation of Crystal Orientation and Phase Distribution of Quasi-2D Perovskite through Synergistic Effect of Additive Doping and Spacer Engineering

**DOI:** 10.1021/acs.inorgchem.4c00335

**Published:** 2024-03-01

**Authors:** Xiao Zhang, Lisanne Einhaus, Annemarie Huijser, Johan E. ten Elshof

**Affiliations:** † Inorganic Materials Science Group, MESA+ Research Institute, University of Twente, 7500 AE Enschede, The Netherlands; ‡ PhotoCatalytic Synthesis Group, MESA+ Research Institute, University of Twente, 7500 AE Enschede, The Netherlands

## Abstract

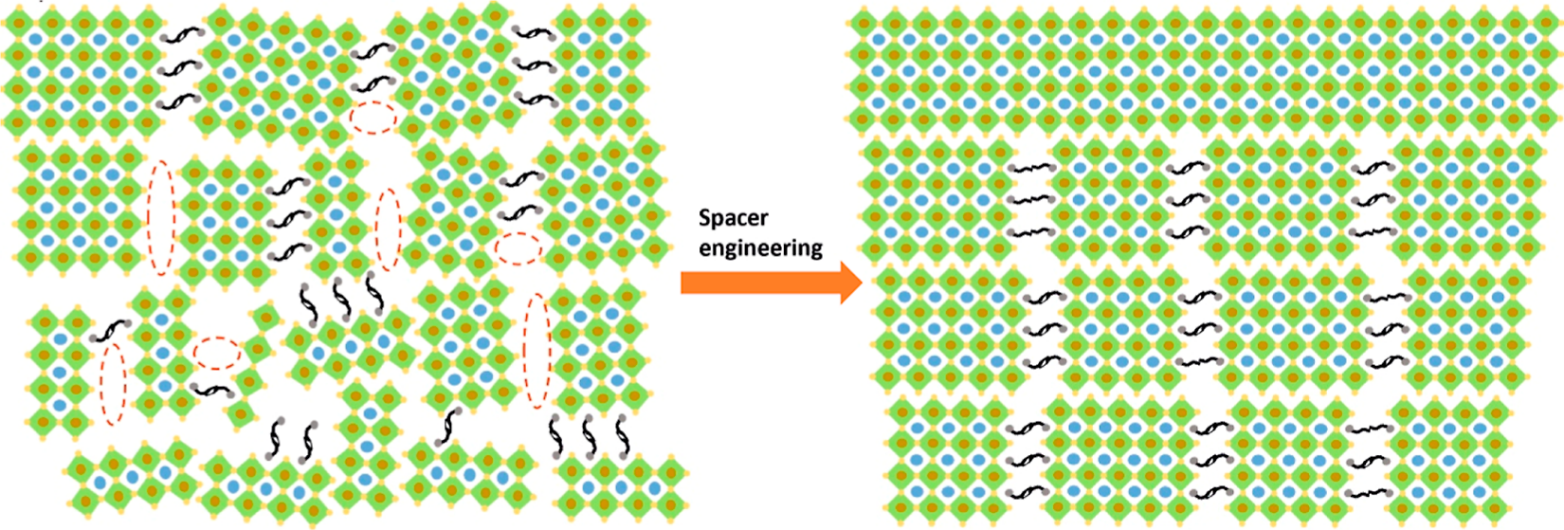

The diammonium precursor 1,4-phenylenedimethanammonium (PDMA) was used as a large organic spacer for the preparation of Dion–Jacobson-type quasi-2D perovskites (PDMA)(MA)_*n*−1_Pb_*n*_I_3*n*+1_ (MA = methylammonium). Films with composition ⟨*n*⟩ = 5 comprised randomly orientated grains and multiple microstructural domains with locally differing *n* values. However, by mixing the Dion–Jacobson-type spacer PDMA and the Ruddlesden–Popper-type spacer propylammonium (PA), the crystal orientation in both the vertical and the horizonal directions became regulated. High crystallinity owing to well-matched interlayer distances was observed. Combining this spacer-engineering approach with the addition of methylammonium chloride (MACl) led to full vertical alignment of the crystal orientation. Moreover, the microstructural domains at the substrate interface changed from low-*n* (*n* = 1, 2, 3) to high-*n* (*n* = 4, 5), which may be beneficial for hole extraction at the interface between perovskite and hole transport layer due to a more finely tuned band alignment. Our work sheds light on manipulating the crystallization behavior of quasi-2D perovskite and further paves the way for highly stable and efficient perovskite devices.

## Abstract 2

A combination of the Dion–Jacobson-type spacer 1,4-phenylenedimethanammonium (PDMA) and the Ruddlesden−Popper-type spacer propylammonium (PA) was used to realize highly crystalline quasi-2D perovskite ⟨*n*⟩ = 5 thin films. Full vertical domain alignment was possible by codoping with methylammonium chloride (MACl). The microstructural domains at the substrate interface changed from low-*n* (*n* = 1, 2, 3) to high-*n* (*n* = 4, 5), which may be beneficial for hole extraction at the interface between a perovskite and a hole transport layer.

## 1 Introduction

Metal halide perovskite has become one of the most promising light harvester and absorber candidates in the last decade, in which 3D methylammonium (MA) lead iodide (MAPbI_3_) perovskite solar cells (PSC) have achieved a rapid rise of power conversion efficiency (PCE) to over 20%, and a record PCE of formamidinium (FA) lead iodide (FAPbI_3_) PSC up to 25.8%, which is comparable to that of conventional Si-based solar cells.^1−11^ However, the low stability of the 3D perovskite phase against moisture, oxygen, heat, and ion migration is one of the main obstacles for its widespread commercialization.^12−22^

**Figure 1:**
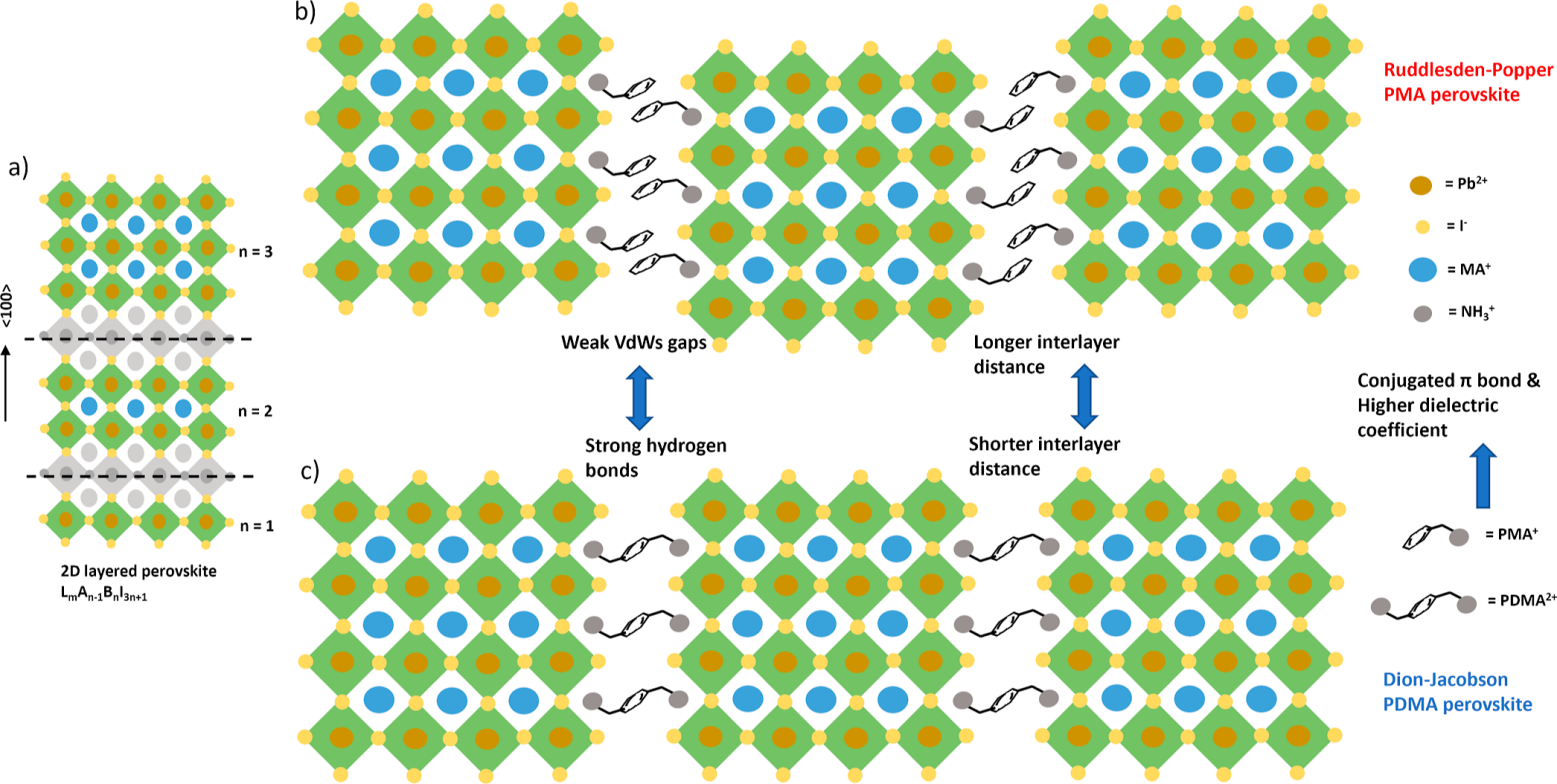
Schematic illustration of the structure of a 2D perovskite. (a) 2D or quasi-2D perovskite by slicing the 3D perovskite in <100> direction with large organic cation spacers. (b) 2D perovskite with Ruddlesden–Popper type monovalent spacer PMA. (c) 2D perovskite with Dion–Jacobson type divalent spacer PDMA.

In order to help solve the urgent instability issue, large organic cation spacers were introduced into the halide perovskite. The organic spacer has an ammonium head that forms hydrogen bonds with iodide ions of the corner-shared [PbI_6_]^4–^ octahedrons, and a long aliphatic chain or a large aromatic ring on the other end, which possesses high hydrophobicity, prevents moisture from penetrating the inorganic layers, and impedes ion migration.^23−34^ The more robust crystal structure also increases the formation energy, and thus improves the stability of the perovskite in the ambient environment.^35^ The ratios of radii of the cations in 3D perovskites are ruled by the Goldschmidt tolerance factor. However, when a large organic spacer is introduced into the 3D perovskite, the sizes of the organic cations do not fit into the octahedral frameworks of the perovskite crystal structure. Instead, it forms a layered crystal structure consisting of layers of corner-shared perovskite octahedrons separated by a monolayer or bilayers of organic cations depending on the ammonium head numbers of the organic spacers. This structure is obtained by “slicing” the 3D perovskite structure along a certain crystal orientation, typically the (100) plane, to maintain the corner-shared octahedrons (Figure 1a).^36−38^ This layered structure is called a 2D perovskite in the field. To be clear, the term “2D perovskite” does not refer to its morphology but is a molecular structure concept. Furthermore, due to the mismatched dielectric coefficient and insulating nature of the organic spacer, 2D perovskites possess an essential quantum-well structure, in which the inorganic layers function as quantum well, while the organic spacer serves as quantum barrier.^39,40^ The light-induced charge carriers cannot be transported into all three principal directions as in a 3D perovskite but are mostly constrained to the inorganic layers. Although the 2D perovskite has a higher chemical and structural stability, the PCE of 2D perovskite solar cells is still lagging behind that of its 3D perovskite counterpart owing to these quantum and dielectric confinement effects.^41−50^ Therefore, knowing how to ameliorate the confinement effect of the 2D perovskite and improve its film quality is vitally important to fabricate a PSC with high and balanced efficiency and stability.

There are three common types of 2D perovskites, namely Ruddlesden–Popper (RP) type, Dion–Jacobson (DJ) type, and alternating-cation-interlayer (ACI) type. The latter one has alternating large organic cations, specifically guanidinium, and small cations; for example, methylammonium in the interlayer between the adjacent inorganic layers, but is not included in this work. The RP and DJ types form a typical chemical formula (L)_*m*_(A)_*n*−1_(B)_*n*_(X)_3*n*+1_, where L represents the large organic mono- or divalent cations, A is the small monovalent cation fitting in the octahedral frameworks (e.g., MA^+^, FA^+^, Cs^+^), B is the divalent metallic cation (e.g., Pb^2+^, Sn^2+^), X is a halide or a mixture thereof (e.g., I^–^, Br^–^, Cl^–^), *m* depends on the type of organic spacer (*m* = 2 for a Ruddlesden–Popper monovalent spacer, *m* = 1 for a DJ divalent spacer), *n* represents the number of inorganic layer between two organic spacers (*n* = 1 for pure 2D perovskite, *n* > 1 for quasi-2D perovskite, *n* = ∞ for 3D perovskite).^51−58^ By changing the composition and *n* value, the bandgap energy of the final film can be tuned, increasing the versatility of the 2D and quasi-2D perovskite family.

In our work, the DJ-type spacer 1,4-phenylenedimethanammonium (PDMA) was used as a large organic cation spacer. The aromatic ring in the spacer can form π-conjugated bonds, which mitigates the dielectric coefficient mismatch with the inorganic counterpart. PDMA also increases the structural rigidity of the framework.^59−67^ The bifunctional cation forms a monolayer that directly bridges the adjacent inorganic layers with strong hydrogen bonds. PDMA interlayers lack the weak van der Waals gaps present in RP spacer layers (Figure 1b). The 2D perovskite with DJ PDMA spacer (Figure 1c) usually possesses a relatively shorter interlayer distance compared to that of a Ruddlesden–Popper spacer with a similar structure, for example, phenylmethylammonium (PMA), which might alleviate quantum confinement effect and potentially facilitate electron tunnelling.^68,69^

For quasi-2D perovskite thin films with a high *n* value (⟨*n*⟩ = 5 in our case) fabricated by the spin-coating method, the microstructural domain within the film is not homogeneously *n* = 5, but comprises of multiple local domains each with different *n* values (e.g., *n* = 2, 3, 4, ..., ∞), the average being ⟨*n*⟩ = 5. The quantum-well structures can grow in all orientations. This random crystal orientation and phase distribution results in a low quality film, with a high defect density and low charge carrier mobility due to the impeded carrier extraction path. Therefore, a large effort has been put into fabricating high quality films of quasi-2D perovskites with a vertical crystal orientation and gradient phase distribution. All the strategies including but not limited to ink and solvent engineering, additive doping, novel spacer design and post-treatment have led to positive outcomes.^70−78^ More recently, researchers combined or mixed RP and DJ spacers as a spacer-engineering approach. For example, Yu et al. combined 1,4-butanediammonium (BDA) and phenylethylammonium (PEA) to realize a quasi-2D MA-based perovskite with *n* = 5, and demonstrated that BDA is located in the crystal grains and PEA is distributed on the surface.^79^ Cheng et al. mixed propane-1,3-diammonium (PDA) and PA to fabricate a quasi-2D FA-based perovskite with *n* = 4, and achieved a PCE improvement of the PSC up to 16.0%.^80^ However, the roles of different spacers and the effect of their mixing on the perovskite film growth mechanism still need a deeper understanding.

Our work aims at tuning the crystal orientation and phase distribution of the quasi-2D perovskite thin film using PDMA and PA spacers, and gaining a deeper understanding of the crystallization behavior and growth mechanism of the quasi-2D perovskite thin film modified by different manipulation strategies. Through synergistic strategies based on both internal precursor manipulation (solvent engineering, additive doping, and spacer engineering) and external processing methods including hot-casting and hot-precursor, the crystal orientation and phase distribution are finely tuned from random to more regulated and preferentially oriented. This synergistic manipulation generates high quality films, facilitating efficient and directional charge transport and preferential energy transfer with a high potential, thus further paving the way for highly stable and efficient perovskite devices.

## 2 Results and Discussion

### 2.1 Dion–Jacobson Type Quasi-2D Perovskite Thin Film

**Figure 2:**
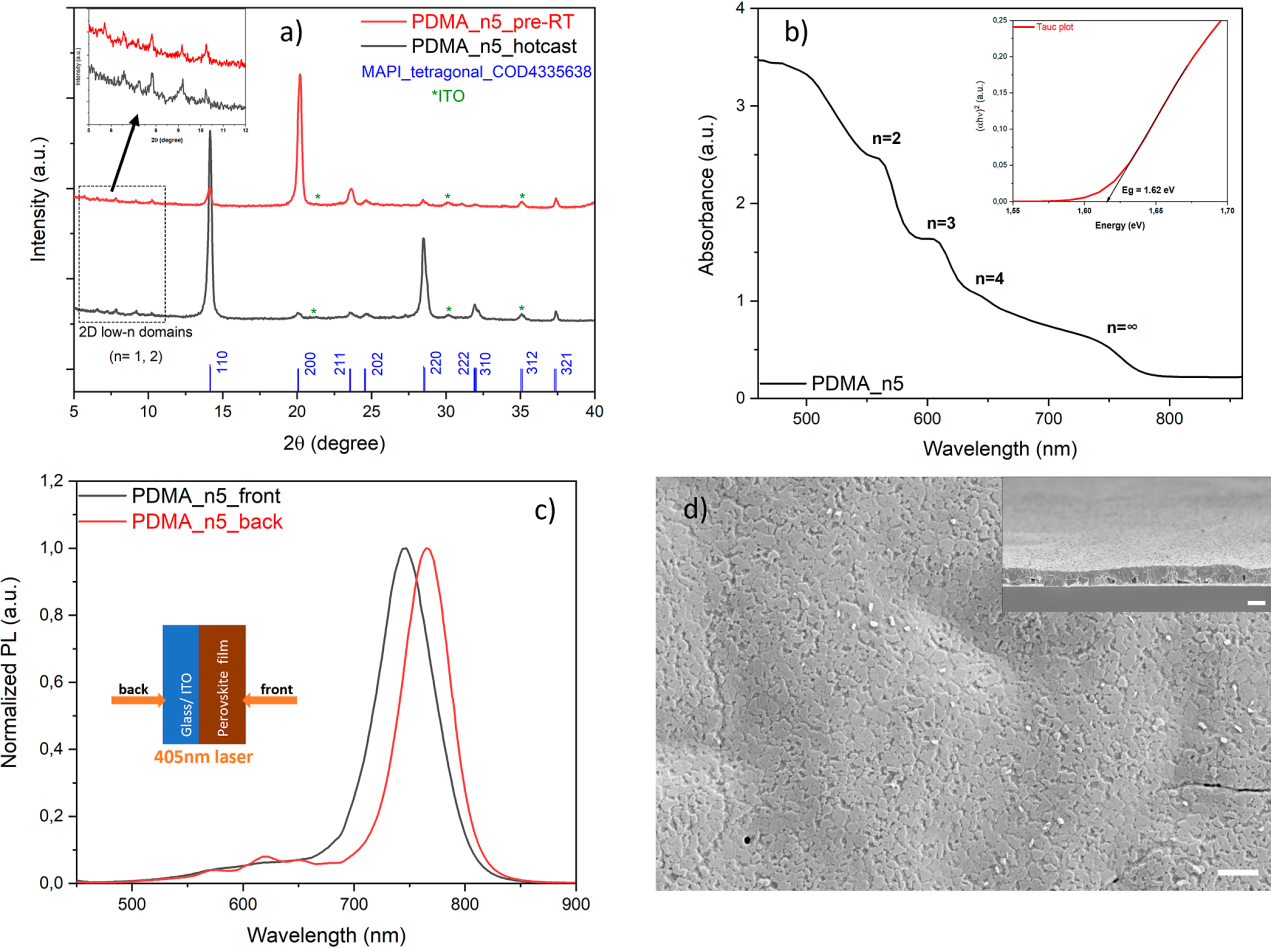
Crystalline orientation, optical properties and morphology of PDMA-based quasi-2D perovskite thin film with ⟨*n*⟩ = 5. (a) Power X-ray diffractograms of quasi-2D perovskite thin films fabricated by the hot-casting method, and made without substrate preheating. Inset displays the magnified plot with 2θ = 5–12°. (b) UV–vis absorption spectra of quasi-2D perovskite thin film (Tauc plot in the inset). (c) Steady-state photoluminescence from front (perovskite) and back (substrate) sides with a near-UV laser of 405 nm (inset shows the laser direction). (d) Surface morphology of the film with a cross-sectional view in the inset, the scale bar is 1 μm.

The DJ PDMA-based quasi-2D perovskite thin film was fabricated by spin-coating the corresponding precursor solution with ⟨*n*⟩ = 5 on a preheated substrate using the hot-casting method. For the detailed fabrication procedure, see the Experimental Section. The mixed solvent dimethylformamide (DMF) and dimethyl sulfoxide (DMSO) was kept constant at DMF/DMSO = 10:1 in volume ratio. This solvent combination is commonly used in solution methods to fabricate perovskite thin films, where DMSO with its higher boiling temperature and stronger coordination bonding with Pb^2+^ forms a stable intermediate complex, which retards the crystallization process.^81−87^ Pb^2+^ is released in the postannealing process during the sequential crystallization following solvent evaporation. Empirically, lower DMF/DMSO ratios have been observed to yield larger grains. However, at the same time, the surface roughness can also increase due to unconstrained growth of few crystalline grains. Thus, the cosolvent system and solvent ratio substantially influence the crystallization process and film morphology and should be fine-tuned. We explored different solvent combinations and ratios including DMF, DMSO, dimethylacetamide (DMAc), and *N*-methyl-2-pyrrolidone (NMP), shown in Figure S1. The combination of a 10:1 volume ratio of DMAc/DMSO showed both (200) and (110) peaks, implying that it has multiple crystal orientations. The combination of 10:1 DMF/NMP, showing low crystallinity, while the surface morphology of the film made from DMF/NMP was very rough. Combined with all data from powder XRD, UV–vis spectra and SEM images in Figures S1 and S2, the combination of 10:1 DMF/DMSO prevailed among all cosolvent combinations and films made. The basic chemical and physical properties of the different solvents used are summarized in Table S1. The crystal phase and orientation are depicted in Figure 2a. Quasi-2D perovskite thin films fabricated either by hot-casting method or without any substrate preheating showed the tetragonal phase of methylammnonium lead iodide (MAPbI_3_) besides the appearance of low-*n* 2D microstructural domains with larger *d*-spacings such as *n* = 1, 2 at 2θ < 10°. Moreover, the data showed different crystal orientations. Especially the film fabricated by hot-casting possesses a dominant (110) peak around 14° and only a small (200) diffraction peak, which indicates vertical alignment with respect to the substrate. In contrast, the predominant (200) peak around 20° and almost absent (110) diffraction in the film without substrate preheating is suggestive of diagonal alignment. There is still discussion about whether (110) or (200) oriented 3D MAPbI_3_ perovskite films are preferred, since both can promote perovskite solar cells of high efficiency.^88^ However, for quasi-2D perovskites with inorganic octahedral layers confined by large organic spacers, a crystal orientation in which the inorganic layers are aligned in the vertical direction to avoid charge transport impedance by organic spacers is preferred for charge transport. Therefore, substrate preheating, or the hot-casting method is imperative to fabricate PDMA-based quasi-2D perovskite thin film with a vertical crystal orientation. Grazing-incidence wide-angle X-ray scattering (GIWAXS) was also measured on pristine PDMA-based DJ perovskite (⟨*n*⟩ = 5). As Figure S9a shows, the film is polycrystalline, as expected, with the two brightest rings at 14 and 28° corresponding to the (110) and (220) peaks, respectively. There are also some minor rings below 10° corresponding to 2D low-*n* domains, which is consistent with the XRD results discussed above.

Although we prepared the precursor solutions by mixing different powders in a stoichiometric way based on the targeted *n* value, here the ⟨*n*⟩ value is still an average number. Besides the absorption onset near the bandgap edge representing its 3D domain with a bandgap of 1.62 eV, there are also excitonic peaks at 559, 604, and 644 nm, representing different 2D low-*n* domains with *n* = 2, 3 and 4, respectively, according to UV–vis spectroscopy (Figure 2b). This means that there are multiple microstructural domains with different *n* values coexisting in the quasi-2D perovskite thin film. Looking at the steady-state photoluminescence (PL) data in Figure 2c, it appears that the different domains tend to form a phase distribution along the film thickness. Laser excitations from the front side of the perovskite film show one dominant emission peak corresponding to the absorption onset. This implies that 3D domains are formed preferentially at the top (air-perovskite) interface. However, in the case of excitation from the back or substrate side, multiple emission peaks at lower wavelengths emerge besides the 3D domain emission peak, implying that more 2D low-*n* domains are formed at the bottom (perovskite-substrate) interface. A broad shoulder at lower wavelength can be seen in the front-side excitation data, implying that partial 2D low-*n* domains are also located toward the top surface, forming an irregular distribution of phases in the vertical direction. The difference between the dominant peaks measured by front and back side excitation is probably due to different incident light entrance paths to the perovskite that go either through air or through a substrate but with a different reflective index. The reabsorption of the hot carriers by different domains with different exciton binding energies may also have changed the dominant emission peak position from front and back side PL. In addition, the composed domains from back and front side could be slightly different due to a difference in the structure. The back side mainly contains 3D domain (*n* = ∞), while the front side contains quasi-3D domain (*n* < ∞). The phase distribution is related to the crystallization process. After spin-coating, the precursor solution deposited on the substrate becomes a sol–gel intermediate state.^89,90^ Upon postannealing, crystallization is initiated at the air–liquid interface where 3D domains crystallize first.^91−94^ After that, the 3D nuclei serve as a template to guide the crystallization process downward into the bulk of the film, and the competition between consumption by large spacers and small cations leads to a phase distribution inside the film. The agglomeration of the large organic spacer at the bottom of the intermediate liquid due to its relatively heavier mass/lower mobility leads to the 2D low-*n* domains formation only toward the end of the crystallization process.^95^ According to a more recent study of a model system of mixed 2D/3D perovskites, 2D domains with different *n* values are actually interspersed in the 3D matrix with a concentration variation in the vertical direction.^96,97^ This explains why emission peaks of 3D domains are both detected by front-side and back-side PL excitation and why the 2D low-*n* domains can also be detected at the top surface.

The morphology of the quasi-2D perovskite thin film is very different from that of the 3D perovskite, where grains and grain boundaries can be clearly seen. In contrast, the quasi-2D perovskite film is more accurately described as chunky material with buried texture due to differences in the local solvent evaporation rate, and top crystalline grains that are not always connected to each other, leaving a rough surface as the SEM image (Figure 2d) shows. From the cross-sectional view, the grown crystals seem to be randomly aligned with the appearance of voids at the perovskite–substrate interface that are probably due to solvent trapping during evaporation in the postannealing stage, particularly DMSO with its a high boiling point.^98^

From the above discussion, it can be concluded that the quasi-2D perovskite thin films grow randomly, although the hot-casting method can promote a preferential vertical crystal orientation. Moreover, the microstructural domains with different *n* values are irregularly distributed in the film. The random crystal orientation and phase distribution impede charge transport and accelerate carrier recombination. In the ideal case of facilitated vertical charge extraction and a preferential energy transfer cascade, vertical crystal orientation and a gradient phase distribution in the film would be present. However, this requires control over the nucleation and crystallization processes both intrinsically and extrinsically. Extrinsic control mainly focuses on changing the processing methods including hot-casting, spinning speed and duration, usage of antisolvent, postannealing temperature and duration, gas quenching, vacuum poling, and so on. Intrinsic control is mainly concerned with tuning the precursor solution itself including the precursor concentration, component mixing ratio (*n* value), solvent composition (solvent type and ratio) as discussed above. The discussion below aims to tune the crystal orientation and phase distribution of quasi-2D perovskite thin film and focuses on other intrinsic film manipulation strategies including additive doping, spacer engineering, and their synergistic effect.

### 2.2 Manipulation Strategies for Tuning Crystal Orientation and Phase Distribution

#### 2.2.1 Additive Doping with MACl

The composition of the precursor solution was a Dion–Jacobson type PDMA-based quasi-2D perovskite with ⟨*n*⟩ = 5 in a mixed DMF/DMSO solvent in a 10:1 volume ratio. The hot-casting method was used to fabricate thin films for all different manipulation strategies. It has been claimed that doping with a chloride salt, for example, methylammonium chloride (MACl), retards the crystallization process, leading to larger grains and regulating the crystal orientation and growth since Cl^–^ can coordinate with Pb^2+^ and also form hydrogen bonds with ammonium (−NH_3_^+^).^99−103^ Although the crystallization process is initiated from the air-perovskite interface and progresses inward, without additives the nuclei in the sol–gel fluid would crystallize homogeneously, leading to a disordered microstructure in the final film. In contrast, the presence of MACl suppresses the crystallization of the sol–gel fluid, thus forcing heterogeneous crystallization directly from top to bottom.^104,105^ In the present investigation, we partially replaced (methylammonium iodide) MAI by MACl as the methylammonium source, while keeping the concentration of methylammonium in the ⟨*n*⟩ = 5 precursor solution constant. The partial replacement of MAI by small fractions of MACl did not result in good quality films with regard to crystal orientation and optical properties (see the XRD and UV–vis absorption data in Figure S3). However, substitution of 75 mol % of all MAI by MACl, further referred to as 0.75 MACl, which results in the overall composition (PDMA) (MA)_4_Pb_5_I_13_Cl_3_, worked well without changing the bandgap.

**Figure 3:**
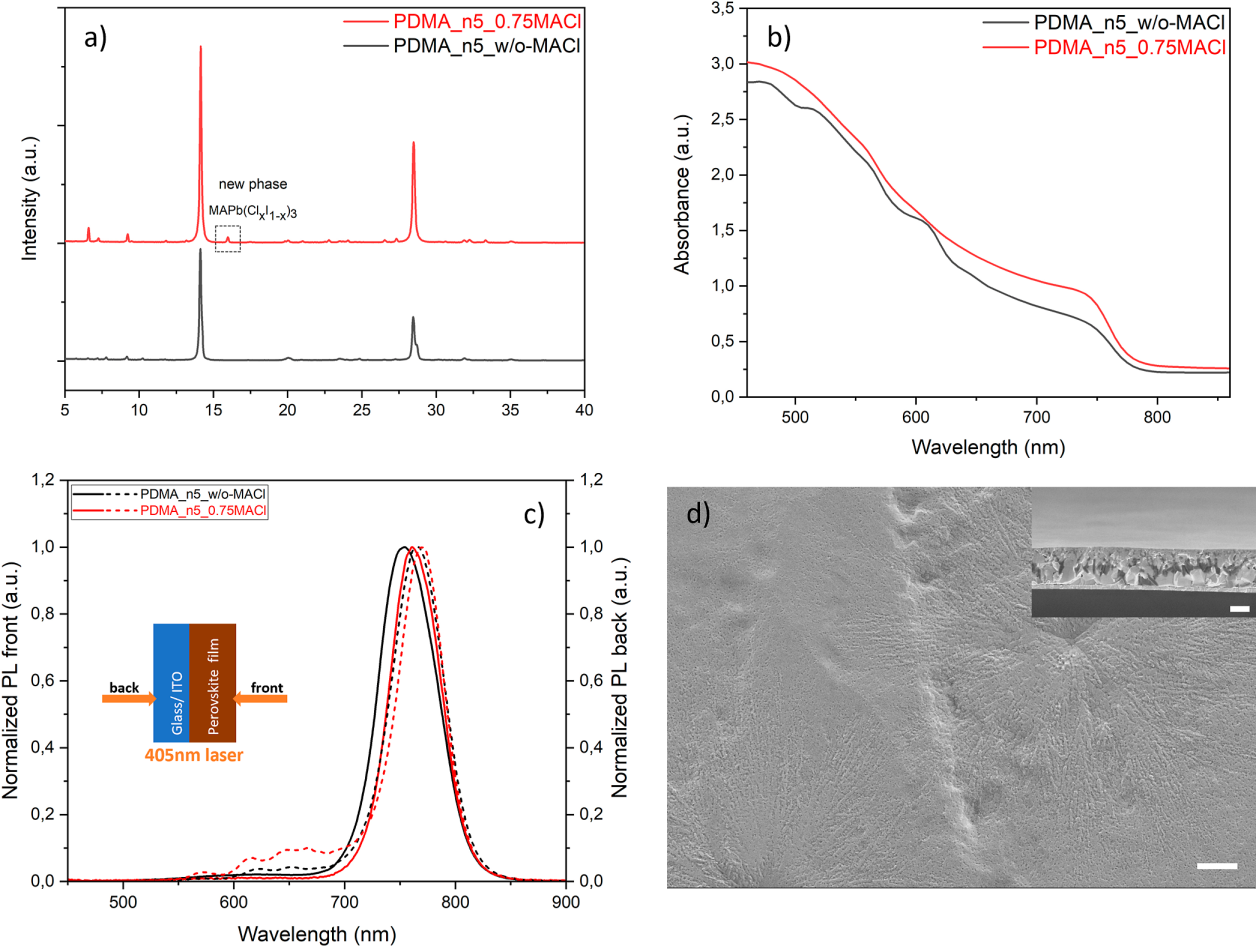
Crystalline orientation, optical properties and morphology of PDMA-based quasi-2D perovskite thin film with ⟨*n*⟩ = 5 without and with the addition of MACl. (a) Power X-ray diffractograms. (b) UV–vis absorption spectra. (c) Steady-state PL from front (perovskite) and back (substrate) sides with a near-UV laser of 405 nm. (d) Surface morphology of the film with 75% substitution of MACl, the scale bar is 2 μm. Cross-sectional view in the inset has a scale bar of 500 nm.

The 0.75 MACl film showed enhanced crystallinity and vertical crystal orientation, as the (110) peak intensity increases, as shown in the XRD data in Figure 3a. A small peak emerged at around 2θ = 16°, consistent with the (100) orientation of methylammonium lead chloride (MAPbCl_3_). The Cl ion has a smaller ionic radius than I^–^, and thus the *d*-spacing of MAPbCl_3_ is smaller than that of MAPbI_3_.^106,107^ Hence, additive doping with MACl results in a mixed phase of MAPb(Cl_*x*_I_1–*x*_)_3_, which is generated along with growth of the perovskite film. On the macroscopic scale, although not fully removed from the film, chloride is distributed homogeneously within the crystalline grains, as shown by the EDS mapping (Figures S4 and S5). The GIWAXS image of the PDMA perovskite with MACl modification (Figure S9b) shows that it is similar to the pristine film, but the 2D low-*n* domains below 10° are a bit more obvious, which is consistent with the XRD data, where the peak intensities below 10° are also higher. In general, the pristine film and the MACl modified film both show disordered orientations, rather than highly vertically aligned.

The UV–vis spectra in Figure 3b show that after adding MACl, the absorbance increased along the whole visible range including the near bandgap edge absorption onset. More interestingly, the excitonic peaks at lower wavelengths representing 2D low-*n* domains are suppressed, although they are still visible in steady-state PL with back-side excitation (Figure 3c), that is, consistent with the observed growth mechanism with which 2D low-*n* domains become located predominantly at the bottom perovskite–substrate interface. Excitons are strongly bonded electron–hole pairs that cannot be easily separated but can be easily recombined and eventually annihilated as heat. Their intrinsic decay is a main cause of the lower efficiency of quasi-2D perovskite solar cells compared to their 3D counterparts. Therefore, MACl doping appears to suppress the photoinduced formation of excitons with a high binding energy and support the formation of more free electrons and holes, which will be extracted sequentially by charge transport layers when integrated into a perovskite solar cell.

The morphology of the 0.75 MACl film was favorable compared to the pristine film without additive doping, as more grains connect each other, leaving a much smoother surface (Figures 3d and S8b). From the cross-sectional view, the buried voids induced by DMSO trapping are absent; all grains are in contact with the substrate, facilitating the contact with the charge transport layer. The vertical growth alignment is improved compared to that of the pristine film without MACl. In conclusion, additive doping with MACl was shown to improve the film quality in terms of regulating the crystal orientation and suppressing the excitons, but there is still room for further improvement, as the laminated structure with small grains in the top layer and large grains in the bottom layer may also be detrimental to charge transport.

#### 2.2.2 Spacer Engineering by Mixing Dion–Jacobson and Ruddlesden–Popper Spacers

The common random crystal orientation and phase distribution in the quasi-2D perovskite thin film originate from its natural quantum-well structure all over the film, the orientation and distribution of which is highly dependent on the crystallization process. A large amount of effort has been put on manipulating the crystallization.^70−77^ However, the outcomes mainly originate from external impact factors e.g. additive, cosolvent, and processing, while the properties of the spacers are usually overlooked. Our theory, on the other hand, emphasizes the function of the large organic spacer, which essentially forms a unique quantum-well structure and makes the crystallization process different from that of the 3D perovskites. However, not all spacer cations that are introduced into the precursor solution are capable of tightly binding the adjacent inorganic octahedral layers in the final film. The DJ-type PDMA spacer is relatively stronger with bifunctional heads and without van der Waals gaps in between as a RP monovalent spacer. Nevertheless, stacking faults of the anchoring and bridging groups of the PDMA spacer onto adjacent [PbI_6_]^4–^ octahedrons causes rotation of the quantum-well structures and the randomness of the whole crystal orientation and phase distribution (Figure 6a). Moreover, the crystallization process is typically so rapid that once a local defect forms during crystallization, the PDMA spacer cannot rearrange itself inside the film, because it has an insufficient number of degrees of freedom and insufficient mobility to do so. Thus, connections to the adjacent octahedra are broken, losing the supramolecular organization and spatial regularity. In contrast, monovalent RP spacers with van der Waals gaps are more tolerant to defects and more flexible with regard to spatial rearrangement.

Our hypothesis is that a DJ divalent spacer can provide only limited regulation of the crystallinity and orientation of quantum-well domains. The hydrogen bonding between the ammonium heads and [PbI_6_]^4–^ octahedrons may not form during crystallization. The missing bridging results in structural defects. However, if a small fraction of a RP spacer is added, the spatial organization of its bilayers can reconnect to the adjacent octahedrons, increase the spatial organization, and lower the number of defects. At the same time, the main structural framework is maintained by a primary DJ spacer. To validate our hypothesis, we conducted the spacer engineering experiments by mixing both the DJ-type PDMA spacer and a RP- type spacer to make up for the PDMA stacking faults and regulate crystal orientation and phase distribution. Based on all of the attempts to mix different types of DJ and RP spacers, we are the first to find that two rules need to be satisfied to mix different types of large organic spacers successfully:

1. The spacers should lead to similar interlayer distances upon forming a 2D perovskite phase by themselves,
2. At least one of the two spacers should have relatively high mobility/lower steric hindrance to organize itself in alignment with the other spacer.

**Figure 4:**
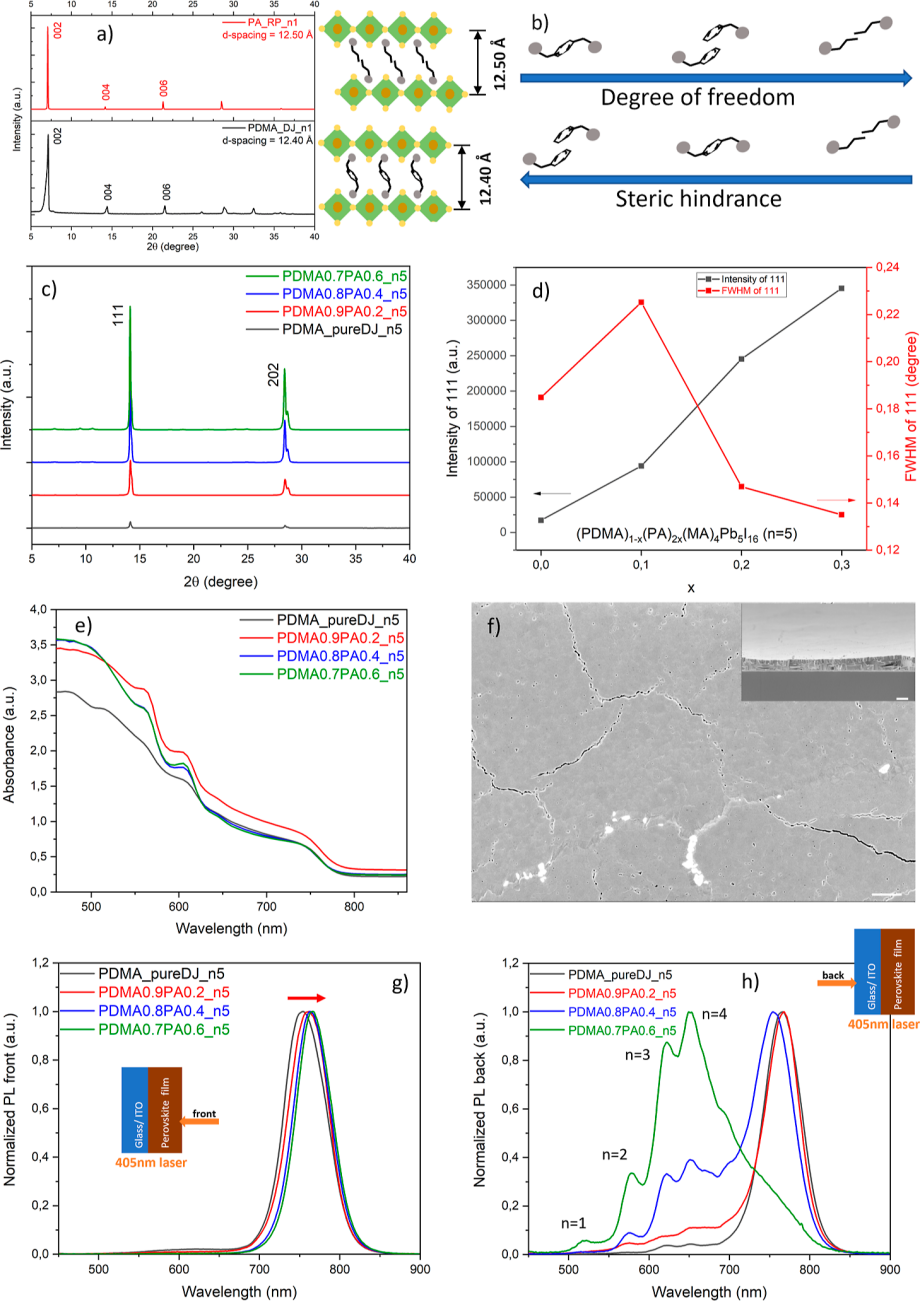
Foundations of spacer engineering and its effect on the crystal orientation and phase distribution. (a) Interlayer distance of 2D perovskite with Dion–Jacobson-type spacer PDMA and Ruddlesden–Popper-type spacer PA. (b) Degrees of freedom and steric hindrance (bulkiness) of different types of spacers (PDMA, PMA and PA). (c) Powder X-ray diffractograms of quasi-2D perovskite thin film (⟨*n*⟩ = 5) with different molar ratios PA/PDMA. (d) XRD intensity and fwhm of (111) peak. (e) UV–vis absorption spectra. (f) Surface morphology of the PDMA0.7PA0.6 film with a cross-sectional view in the inset, the scale bar is 1 μm. (g,h) Steady-state PL data from front (perovskite) and back (substrate) side, respectively, with a near-UV laser of 405 nm (inset shows the illumination direction).

The combination of a PDMA spacer and PA spacer seems to be one of the few candidates that have an optimized result. We also tried other combinations, for example, PDMA and PMA spacers, but they failed in fixing the missing bridging in the interlayer. In Figure 4a,b, two RP spacers are shown for comparison, namely PA with a short aliphatic chain and PMA with a large aromatic ring. PMA is a bulky molecule, causing large steric hindrance. The monovalent spacer forms a bilayer between the inorganic octahedra, and the large benzyl ring expands its spatial volume so that it does not match the length of the DJ PDMA spacer with a similar aromatic ring. Moreover, by forming a 2D perovskite with alternating inorganic layers and organic spacers, we can calculate the interlayer distance of (PMA)_2_PbI_4_ to be 14.37 Å, so 16% larger than that of (PDMA)PbI_4_ (12.40 Å). Mixing of PDMA and PMA spacers showed no improvement in crystallization except for an irregular phase distribution with 2D low-*n* domains located on both the top and bottom sides (Figure S6). In contrast, when PA was used as the cospacer next to PDMA, it not only has a good match with the interlayer distance (12.50 Å) of PDMA (Figure 4b), but it also has lower steric hindrance due to its short aliphatic chain length and its higher rotation flexibility, thereby following the two fundamental rules for mixing different types of spacers stated above.

As we know, the DJ PDMA-based quasi-2D perovskite precursor solution of ⟨*n*⟩ = 5 is prepared in the stoichiometric way following reaction 1 below, while the RP PA-based analogue is prepared by reaction 2. The precursor solution with mixed spacers is then prepared by mixing different molar ratios of PDMA and PA spacers following reaction 3, which is the sum of reactions 1 and 2 for the case where PAI molecules represent a molar fraction *x* of all spacer molecules PA and PDMA.
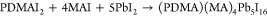
1
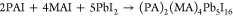
2
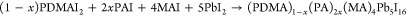
3

A film with a PA fraction *x* is labeled PDMA(1 – *x*)PA(2*x*), so a film with *x* = 0.1 is designated below as PDMA0.9PA0.2, etc. With increasing fraction *x*, the crystallinity and vertical crystal orientation was profoundly enhanced, as evidenced by the increasing intensity and decreasing full width at half-maximum (fwhm) of the (111) peak in XRD data in Figure 4c,d. It is noted that for the 3D tetragonal MAPbI_3_ perovskite phase, the peaks at around 14 and 28° are usually labeled as (110) and (220), respectively, while the same peaks in a quasi-2D perovskite thin film are usually labeled as (111) and (202), due to the fact that the inorganic layers are slightly differently aligned with respect to the substrate due to the separation by the large organic spacers.^77,108^ We noticed that the two peaks in the quasi-2D perovskite films actually contain a peak splitting or doublet. The double XRD peaks at around 14.1 and 14.3° correspond to the (002) plane of the tetragonal 3D MAPbI_3_ perovskite phase and the (111) plane of a quasi-2D perovskite phase, respectively. Similarly, the double peaks around 28.4 and 28.7° correspond to the (004) plane of the tetragonal 3D MAPbI_3_ perovskite phase and the (202) plane of the quasi-2D perovskite phase, respectively. This also implies that both 3D and quasi-2D microstructural domains are present in the films. In Figure S9c, the GIWAXS data show that, in contrast to the pristine PDMA and PDMA with MACl modified films, the rings in the perovskite film with PDMA and PA mixed spacers (PDMA0.7PA0.6) are brighter and sharper, which indicates that the crystallinity is higher and the crystals are more preferentially oriented. The rings indicate the presence of texture in the polycrystalline film. The reason is the missing bridging fixation and the crystal orientation regulation by mixing the two types of spacers, as elaborated above.

The steady-state PL signal recorded by the excitation from the front (perovskite) side of the film shows one dominant emission peak, confirming the presence of 3D domains at the top (Figure 4g). The maximum of the emission peak is slightly red-shifted from 755 to 766 nm with an increasing PA/PDMA ratio. This implies the presence of even more 3D domains on the front side, which is usually an indication of defect reduction and film quality improvement. The intensity of the shallow peak at low wavelength decreases from pure PDMA-based film to PDMA0.9PA0.2 film and is not present in PDMA0.8PA0.4 and PDMA0.7PA0.6 films. This means that the presence of 2D low-*n* domains located at the top side of the film is suppressed, and thus that the phase distribution throughout the film is more regulated. However, when exciting the film from the back (substrate) side, the phase distribution seems to be very different. The 3D domain peak blue-shifts when the composition changes from pure PDMA via PDMA0.9PA0.2 to PDMA0.8PA0.4 film, which indicates that the total volume fraction of the 3D domain is smaller. The signals from the 2D low-*n* domains increased simultaneously until the composition PDMA0.7PA0.6, where no 3D domain peak was detected. Instead, the 2D low-*n* domains where *n* = 1, 2, 3, and 4 corresponding to the emission peaks at 519, 579, 622, and 652 nm, respectively, are dominant at the perovskite–substrate interface (Figure 4h). It demonstrates that 2D fragments are not interspersed throughout the 3D network but that the 2D domains are at the bottom, spatially separated from the 3D domains at the top. It has been suggested that the 2D low-*n* domains are inclined to align horizontally, especially *n* = 1 and 2.^109^ However, the PDMA0.7PA0.6 film shows more 3D domains and the highest vertical crystal orientation intensity among the four investigated PA/PDMA ratios. This seemingly contradictory result is supported by the cross-sectional SEM image of a PDMA0.7PA0.6 film, where both vertical and horizontal crystal orientations are present (Figure 4f). The cracks probably result from internal stresses that built up following spatial contraction and densification by both vertical and horizontal alignment processes. The pure PA-based film has a similar surface morphology with cracks and a high degree of vertical alignment (Figure S7c). Therefore, even the presence of a small amount of PA spacer in a PDMA-based precursor solution influences the morphology of the final film drastically. The high light absorption of PDMA0.9PA0.2 is probably due to its lower number of cracks and its denser and more compact film structure compared with the other ones (Figures 4e and S7a).

Hence, it seems that the combination of the DJ-type bifunctional PDMA spacer and the RP-type monovalent PA spacer leads to improved crystallization because of their matching interlayer distance and because PA has lower steric hindrance than PDMA and other benzyl-ring-based spacers like PMA. Our hypothesis that any missing bridging to adjacent inorganic octahedrons by some of the PDMA spacers due to their low degrees of freedom and large steric hindrance is compensated by the presence of the smaller PA spacer with its ability of rearrangement (Figure 6b). The crystal orientation can be regulated in both vertical and horizontal directions, which is consistent with the changes in its phase distribution throughout the film. This crystal growth manipulation strategy opens up a new path to tune the crystal orientation and phase distribution in quasi-2D perovskite thin films. The coexistence of vertical and horizontal alignment may impede charge transport in the vertical direction.^93,110−112^ Therefore, spacer engineering does not end the investigation but inspires us to implement further modifications, in order to form a quasi-2D perovskite film toward completely vertical orientation and ordered phase gradient in the next stage.

#### 2.2.3 Synergistic Effect of Additive Doping, Spacer Engineering and Processing Methods

**Figure 5:**
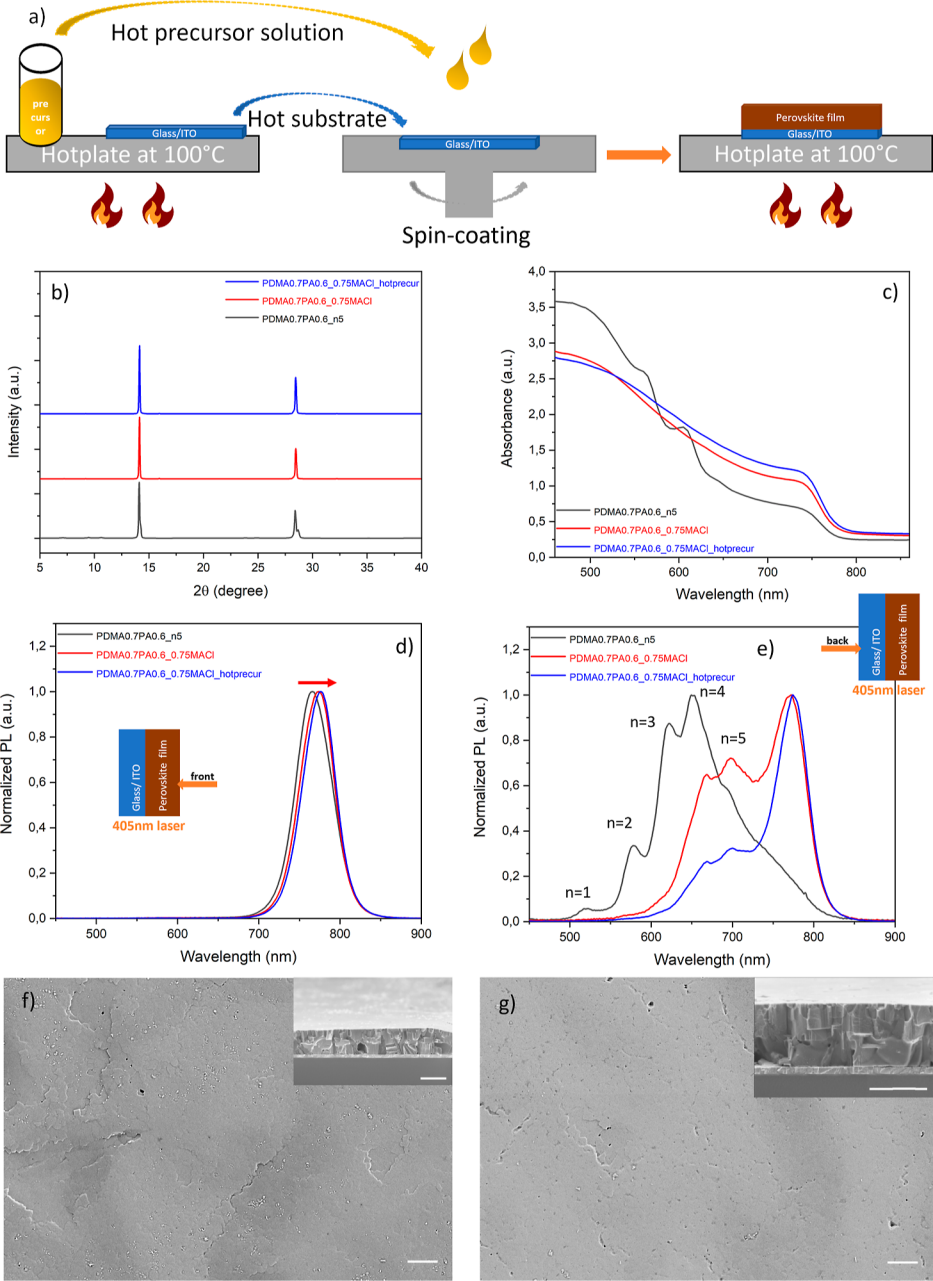
Synergistic effect of additive doping, spacer engineering, and processing methods. (a) Schematic illustration of combined processing methods of hot-casting and hot-precursor. (b,c) PoVAwer X-ray diffractograms and UV–vis absorption spectra of quasi-2D perovskite thin film with ⟨*n*⟩ = 5, namely a mixed-spacer film (PDMA0.7PA0.6), a film with combined spacer engineering and additive doping (PDMA0.7PA0.6_0.75MACl), and a PDMA0.7PA0.6_0.75MACl film with extra hot-precursor processing. (d,e) Steady-state PL from front (perovskite) and back (substrate) side, respectively, with a near-UV laser of 405 nm (inset shows the laser direction). (f,g) Surface morphology of the PDMA0.7PA0.6_0.75MACl film and PDMA0.7PA0.6_0.75MACl + hot-precursor film with cross-sectional views in the insets. The scale bars are all 1 μm.

We explored the combination of spacer engineering and the additive doping approach discussed above and investigated its effect on the perovskite film growth. We adopted a hot-precursor processing method, in addition to hot-casting. In the hot-casting method, the preheated substrate is quickly transferred from the hot plate to the chuck of the spin-coater before dripping the cool precursor solution. However, the temperature of the substrate will inevitably drop during the transfer, leaving a very narrow processing window for precursor deposition. However, if a preheated precursor solution is dropped simultaneously (Figure 5a), the processing window is expanded, thereby reducing the thermal strain at the perovskite–substrate interface, lowering the energy barrier of heterogeneous nucleation, facilitating spontaneous crystallization, and resulting in enlarged grain size and reduced defects.^113,114^

We fixed the composition in the mixed-spacer film at PDMA0.7PA0.6. Additive doping was included, substituting 75 mol % of MAI by MACl as the methylammonium source, which is labeled further on as PDMA0.7PA0.6_0.75MACl, the equivalent of (PDMA)_0.7_(PA)_0.6_(MA)_4_Pb_5_I_13_Cl_3_. As shown in Figure 5b, the vertical crystal orientation was enhanced upon introduction of MACl, but it did not form a detectable MAPb(Cl_*x*_I_1–*x*_)_3_ phase, as no peak around 16° was observed. Peak splitting was slightly lower, implying strain release was induced by octahedron tilting. From the UV–vis spectrum is Figure 5c, the excitonic peaks representing the 2D low-*n* domains were suppressed as expected, due to the effect of MACl doping, and the absorbance above 600 nm was increased until the near-IR region including the absorption onset, which is dominated by the absorption of 3D domain at the bandgap edge. The trend in absorption was further confirmed by the steady-state PL data upon excitation from the front (perovskite) side in Figure 5d, showing that the emission peak red-shifted from 766 nm for the PDMA0.7PA0.6 film to 773 nm for PDMA0.7PA0.6_0.75MACl, which is attributed to the larger volume fraction of 3D domains at the front side. The steady-state PL data from the back (substrate) side excitation show that the contributions from 2D low-*n* domains (*n* = 1, 2, 3) are diminished, while *n* = 4 and 5 domains corresponding with the emission peaks at 668 and 698 nm, respectively, are present at the bottom, embedded in the 3D matrix with its peak maximum reappearing at 770 nm (Figure 5e). The film grew even denser and more compact, compared to the PDMA0.7PA0.6 film (Figure 5f), and no obvious cracks can be seen.

Hot-precursor processing of the PDMA0.7PA0.6_0.75MACl film shows even more enhanced vertical crystal orientation and light absorption than the film without extra hot-precursor processing as displayed in Figure 5b,c. The emission peak of the 3D domains from front side excitation was further red-shifted to 777 nm (Figure 5d), which is attributed to a better film quality with fewer defects. The concentration of *n* = 4 and 5 domains at the back side is small compared to the volume fraction of 3D domains, which red-shifted to 776 nm (Figure 5e). These 3D domains near the substrate interface promote preferential charge transport and energy transfer. The GIWAXS results displayed in Figure S9d,e also show that both the PDMA0.7PA0.6_0.75MACl film with only hot-casting fabrication and the film with both hot-casting and hot-precursor processing exhibit rings that are even brighter and sharper compared to the PDMA0.7PA0.6 film without MACl doping, implying that the crystallinity is even more enhanced, and the crystal orientation is even further regulated and is fully vertically aligned. The intensities of the 2D low-*n* domains are lower than in the PDMA0.7PA0.6 film, which is consistent with the UV–vis absorption data and the steady-state PL data, where low-*n* domains were diminished, and high-*n* domains and 3D domain were dominant, as discussed above. The film surface also became smoother with fewer defects (Figures 5g and S8e). From the cross-section view, the film shows large monolithic grains throughout the whole film thickness, facilitating charge extraction, particularly in the vertical direction.

In the inverted p–i–n solar cell configuration, the photogenerated electrons from the perovskite layer are extracted from low-*n* (large bandgap) to high-*n* (narrow bandgap) domain from bottom to top side, while holes are transported in the opposite direction. So when the 2D low-*n* domains at the bottom are mainly *n* = 1 and 2, the large bandgap induced valence band maximum uplifting will impede downward hole extraction, while if we tune the 2D low-*n* domain at the bottom side to be *n* = 4 and 5, the better matched energy level alignment should theoretically facilitate hole extraction.^87^

**Figure 6:**
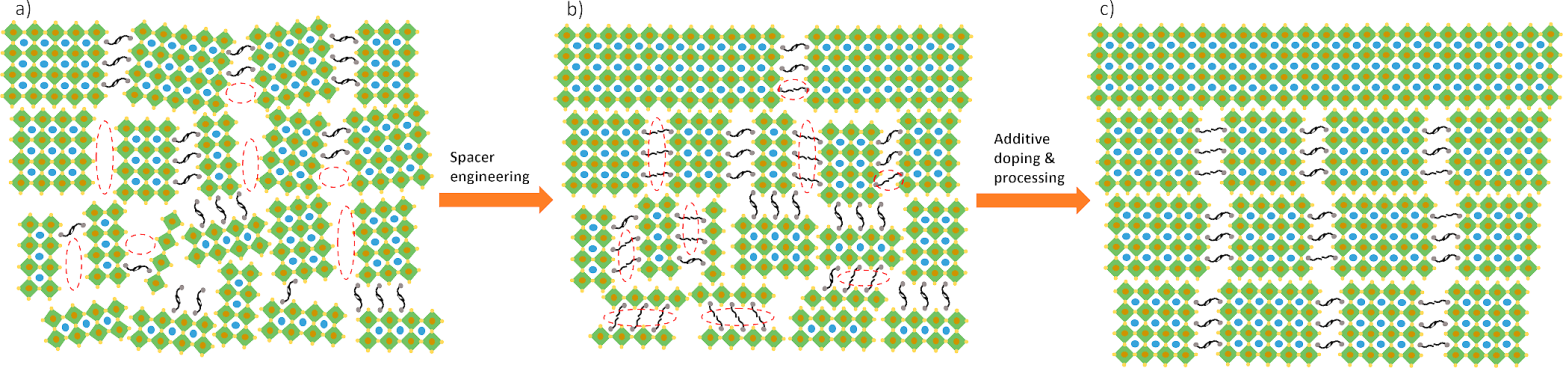
Schematic illustration of crystal orientation and phase distribution in quasi-2D perovskite thin film of *n* = 5. (a) Pure PDMA film, the red dashed ellipses represent the missing bridging left by the bulky PDMA spacer, causing low crystallinity, lack of orientation, and distribution. (b) Mixed PDMA–PA film, where the red dashed ellipses represent the interlayer connections formed up by the PA spacer. The structure is regulated simultaneously in vertical and horizontal directions. (c) Film with the combination of additive doping and spacer engineering, facilitating the whole structure into a preferential vertical crystal orientation and gradient phase distribution.

In summary, we successfully tuned the crystal orientation and phase distribution of quasi-2D perovskite thin films from random to regulated to preferential (Figure 6c). Especially the synergistic effects of additive doping, spacer engineering, and hot precursor processing enhanced the vertical crystal orientation and light absorption, improved the film quality and vertical growth alignment, and also promoted the preferential phase distribution by tuning particularly the 2D domains at the bottom side from low-*n* to high-*n*, thereby facilitating better charge transport pathways.

## 3 Conclusions

For DJ-type PDMA-based perovskite thin film with *n* = 5, random growth and irregular microstructural domain distribution were observed. Additive doping with MACl led to enhanced light absorption, suppressed excitonic absorption induced by 2D low-*n* domains, and a smoother surface, but the improvement was limited. Mixing of the two different types of spacers (DJ type PDMA and RP-type PA) generates films with well-defined crystallinity and regulated crystal growth by promoting both vertical crystal orientation of high-*n* domains and horizontal alignment of 2D low-*n* domains. The combination of additive doping, spacer engineering, and processing methods led to control over the crystal orientation and phase distribution. The step-by-step tuning shed light on the growth mechanism of quasi-2D perovskite thin film. The synergistic effect not only enhanced the vertical crystal orientation and light absorption and improved the film quality but also promoted the preferential phase distribution by tuning particularly the 2D domains at the bottom from low-*n* to high-*n*, potentially facilitating better charge extraction across the interface of the perovskite and charge transport layer.

## 4 Experimental Section

### 4.1 Materials

Lead(II) iodide (PbI_2_, 99%), methylammonium iodide (MAI, ⩾99%), *N*,*N*-dimethylformamide (DMF, 99.8%), dimethyl sulfoxide (DMSO, ⩾99.9%), *p*-xylylenediamine (PDMA, 99%), hydroiodic acid 57% (HI, for synthesis), and diethyl ether (⩾99.9%) were purchased from Sigma-Aldrich. Propylamine (PA, ⩾99.0%) were purchased from Fluka. The above-mentioned were used as received without further purification. Methylammonium chloride (MACl, for synthesis) was purchased from Sigma-Aldrich, and was further dried in vacuum oven before use.

### 4.2 Synthesis of PDMAI_2_ Power

First 3.485 g of PDMA was dissolved in EtOH with stirring. Then 7.37 mL of HI was added and heated in 100 °C oil bath until all precipitates were solidified and all of the liquid was evaporated. The yellowish clay-like precipitates were purified with diethyl ether. This was followed by transferring them into a Petri dish. Finally, the precipitates were dried in a 60 °C vacuum oven for 3 days.

### 4.3 Synthesis of PAI Powder

First 13.20 mL of HI was added into 8.29 mL of PA, and then heated in 100 °C oil bath with continuous stirring, until all precipitates were solidified and all of the liquid was evaporated. The precipitates were purified with a large amount of diethyl ether until they turned white. This was followed by transferring them into a Petri dish. Finally, the precipitates were dried in a 60 °C vacuum oven for 3 days.

### 4.4 Preparation of Precursor Solutions

For ⟨*n*⟩ = 5 DJ precursor solution, dissolve PDMAI_2_, MAI, and PbI_2_ powders in a stoichiometric molar ratio of 1:4:5 with a Pb^2+^ concentration of 1 M in the mixed solvent DMF/DMSO = 10:1. For ⟨*n*⟩ = 5 mixed DJ and RP precursor solution, dissolve PDMAI_2_, PAI, MAI, and PbI_2_ powders in a stoichiometric ratio of (1 – *x*):2*x*:4:5 with a Pb^2+^ concentration of 1 M in the mixed solvent DMF/DMSO = 10:1 v/v. For the additive doped precursor solution with the addition of MACl, MAI was replaced with MACl as the methylammonium resource still in a stoichiometric ratio of MAI/MACl = (1 – *y*):*y*. For instance, for the most sophisticated composition, the powders are mixed in the ratio of PDMAI_2_/PAI/MAI/MACl/PbI_2_ = (1 – *x*):2*x*:4(1 – *y*):4*y*:5.

### 4.5 Fabrication of Quasi-2D Perovskite Thin Films

First the ITO-covered glass substrate was cleaned with detergent, DI water, acetone, and IPA sequentially in an ultrasonic bath. Then the substrate was treated by O_2_ plasma. After that, the substrate was transferred to the N_2_ glovebox and preheated on the 100 °C hot plate for 10–15 min beforehand. After the substrate was transferred quickly to the chuck of the spin-coater, 60 μL of precursor solution was dynamically spin-coated on the hot substrate (hot-casting) with a stepwise spinning program of 1500 rpm for 15 s and 4000 rpm for 20 s. The final film was formed by postannealing on a 100 °C hot plate for 10 min.

### 4.6 Characterizations

The powder XRD was measured by a Panalytical X’pert Pro Powder diffractometer with Cu Kα1. The UV–vis absorption spectra was measured by UV/vis/NIR Spectrometer Lambda 950 (PerkinElmer) transmittance (*T*) mode, after which the absorbance (*A*) was calculated by *A* = 2 – log(*T*). Steady-state photoluminescence was measured by the Blue-Wave Spectrometer from StellarNet Inc. with a 405 nm laser from MatchBox series and a FGL435S color filter from Thorlabs. The SEM (point resolution) is measured by a Zeiss Merlin HR-SEM equipped with an EDX (spatial resolution).

## Supporting Information Available

Additional experimental data (XRD, SEM–EDX, AFM, and UVV) and including solvent data (PDF)
