# Supplementary material for: Manipulation of Crystal Orientation and Phase Distribution of Quasi-2D Perovskite through Synergistic Effect of Additive Doping and Spacer Engineering
Source: Inorg Chem. 2024 Mar 1;63(11):5246–59. doi: 10.1021/acs.inorgchem.4c00335 (PMC10951954; doi:10.1021/acs.inorgchem.4c00335)
Supplement: Supplementary file 1 — ic4c00335_si_001.pdf [file ic4c00335_si_001.pdf]

Supporting information

Manipulation of Crystal Orientation and Phase Distribution of Quasi-2D Perovskite through Synergistic Effect of Additive Doping and Spacer Engineering

Xiao Zhang[1], Lisanne Einhaus[2], Annemarie Huijser[2], Johan E. ten Elshof[1]

[1] Inorganic Materials Science Group, MESA+ Research Institute, University of Twente, 7500 AE Enschede, the Netherlands; [2] PhotoCatalytic Synthesis Group, MESA+ Research Institute, University of Twente, 7500 AE Enschede, the Netherlands

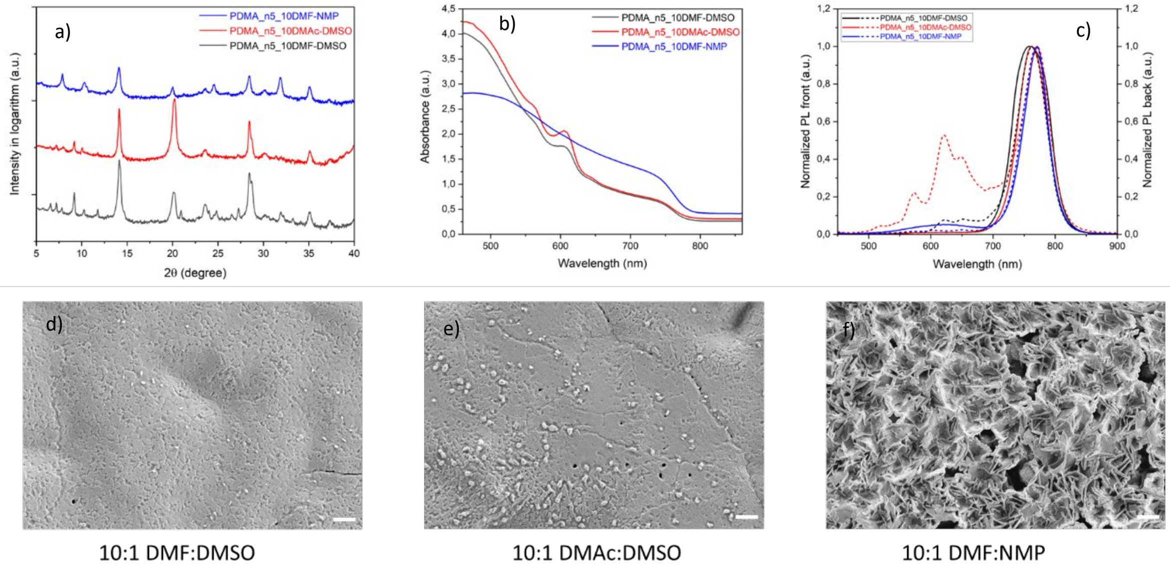

Figure S1. Effect of three different cosolvents (DMF:DMSO; DMAc:DMSO; DMF:NMP) on crystallinity crystal orientation, optical properties and surface morphology with a fixed volume ratio of 10:1. a) Power X-ray diffractograms. b) UV-Vis absorption spectra. c) Steady-state PL from front and back excitation. d-f) SEM images of quasi-2D perovskite films fabricated by solvent combination of DMF:DMSO, DMAc:DMSO, DMF:NMP, respectively. The scale bar is 1 µm.

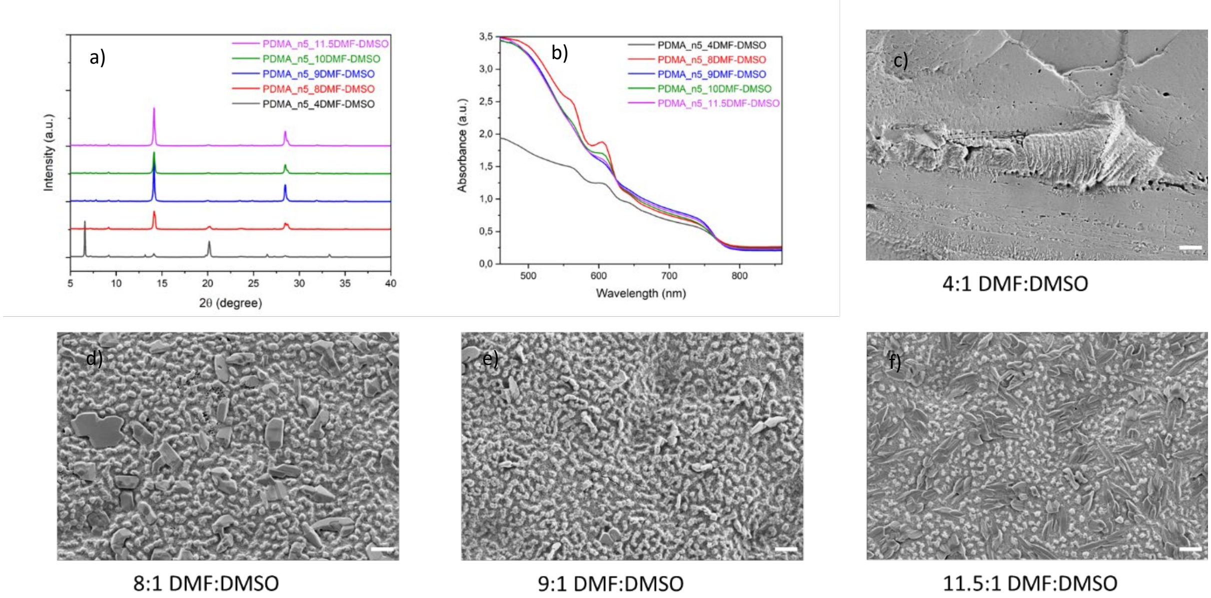

Figure S2. Effect of different solvent ratios on crystal orientation, optical properties and surface morphology with a fixed solvent combination of DMF and DMSO. a) Power X-ray diffractograms. b) UV-Vis absorption spectra. c-f) SEM images of quasi-2D perovskite films fabricated by different ratios of DMF and DMSO of 4:1, 8:1, 9:1 and 11.5:1, respectively. The scale bar is 1 µm.

Table S1. Different solvents and their chemical structure, boiling point and dipole moment.

Solvent	Chemical structure	Boiling point	Dipole moment
DMF	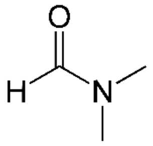	153°C	3.86D
DMSO	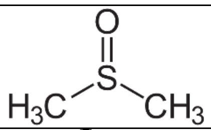	189°C	4.00D
DMAc	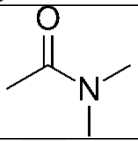	165°C	3.72D
NMP	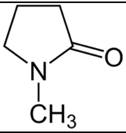	203°C	4.09D

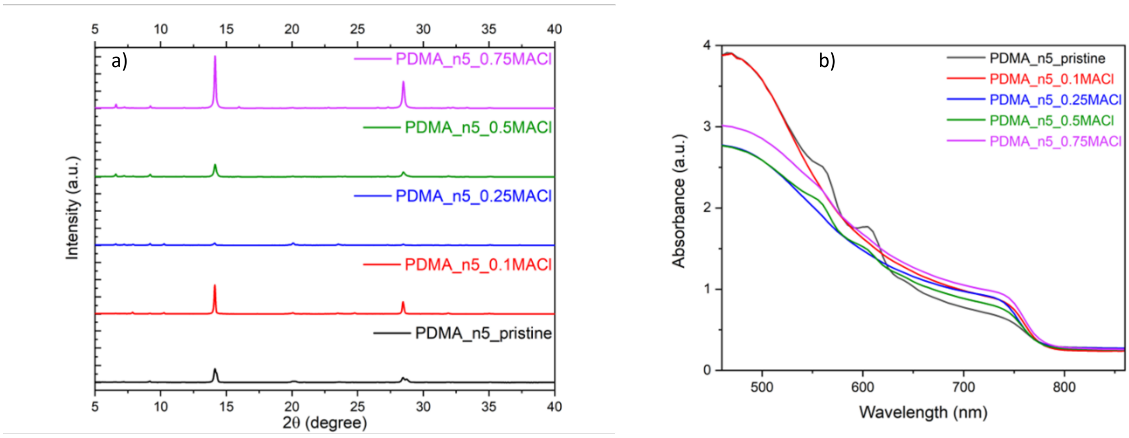

Figure S3. a) Power X-ray diffractograms and b) UV-Vis absorption spectra of quasi-2D perovskite thin film with different additive (MACl) doping ratio.

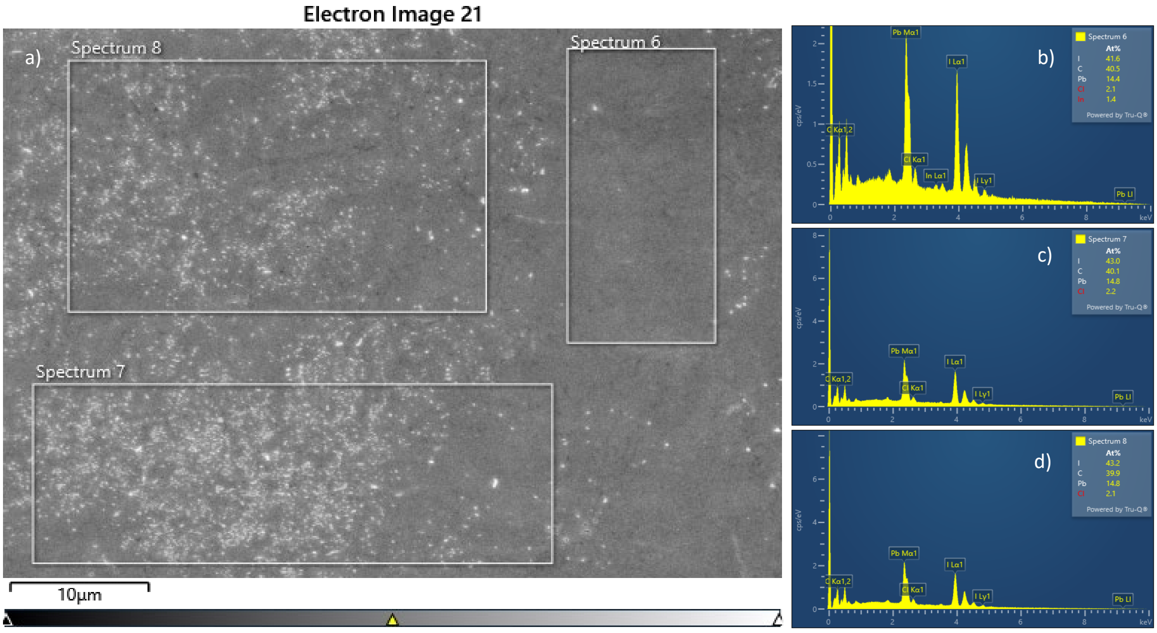

Figure S4. a) top view SEM of PDMA0.7PA0.3_0.75MACl quasi-2D perovskite thin film. b-d) EDS results of three selected areas of a), displaying the elemental ratio.

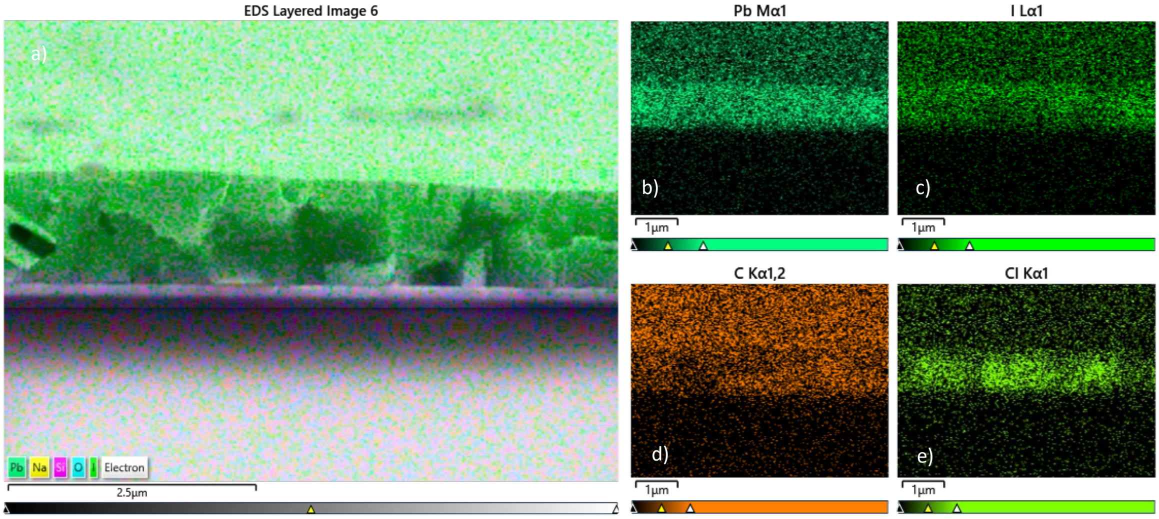

Figure S5. a) cross-sectional view SEM of PDMA0.7PA0.6_0.75MACl quasi-2D perovskite thin film. b-e) EDS map of the corresponding area of a), displaying the elemental distribution.

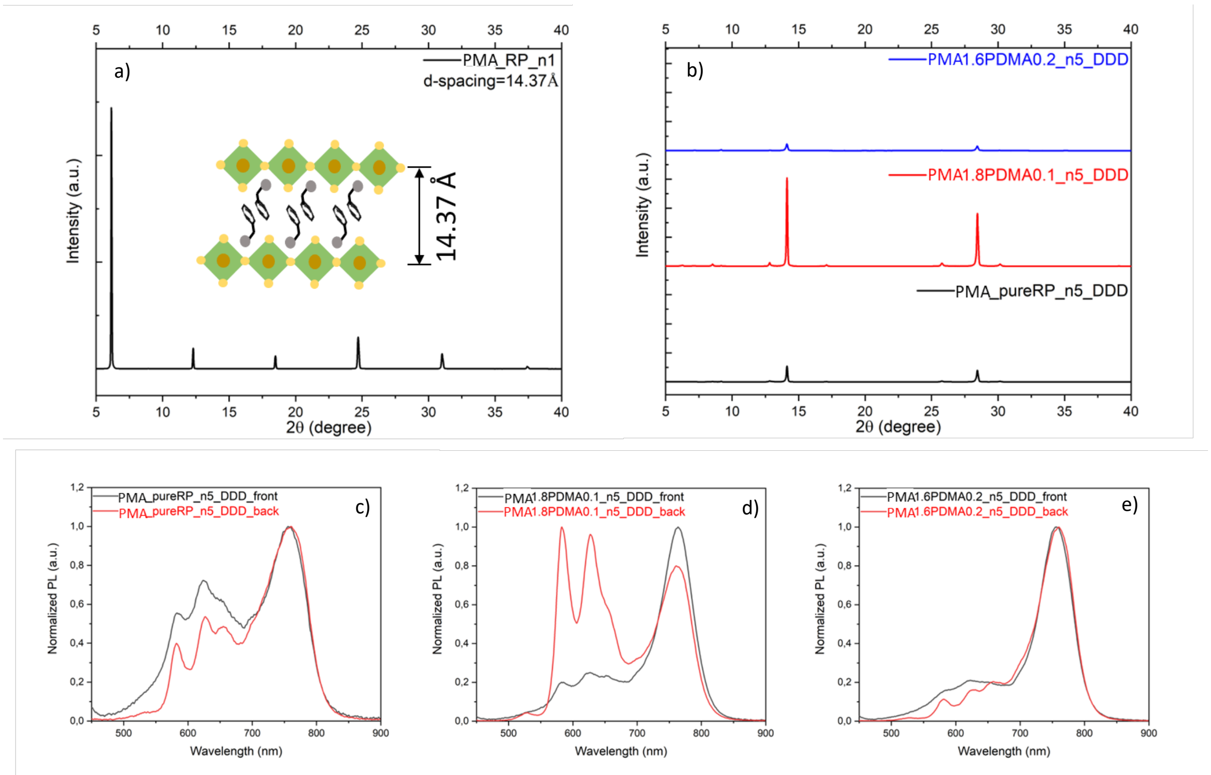

Figure S6. Spacer engineering using PMA and PDMA spacer. a) Power X-ray diffractogram of pure PMA film with an interlayer distance of 14.37 Å. b) Power X-ray diffractograms of quasi-2D perovskite thin film with <*n*>=5 with different ratios of mixed spacers. PMA_pure-RP film is quasi-2D perovskite film with only Ruddlesden-Popper PMA spacer, PMA1.8PDMA0.1 film is the one with mixed PMA and PDMA spacer in the ratio $((PMA)_2)_{0.9}(PDMA)_{0.1}$, similar to the labelling of PMA1.6PDMA0.2 film. c-e) Steady-state PL from front and back side laser excitation (405nm) of the corresponding films with different spacer mixing ratios.

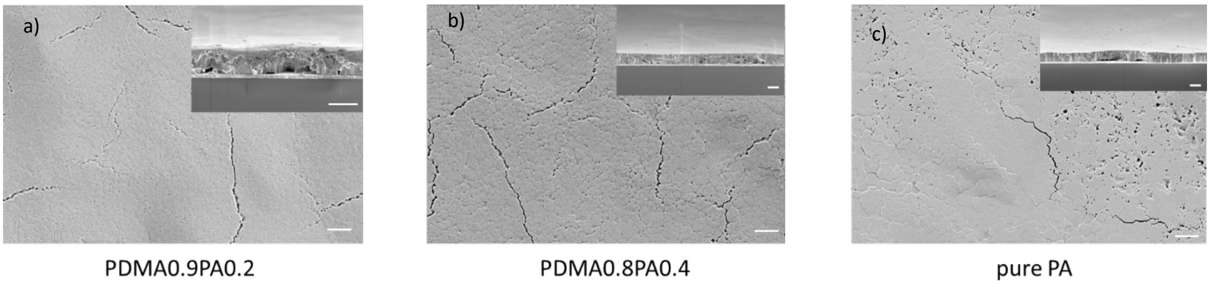

Figure S7. Surface morphology of quasi-2D perovskite thin films with *n*=5 with different mixing ratios of Dion-Jacobson PDMA spacer and Ruddlesden-Popper PA spacer. a) PDMA0.9PA0.2 film. b) PDMA0.8PA0.4 film. c) Pure PA film. Insets are the cross-section views. The scale bars are all 1 µm.

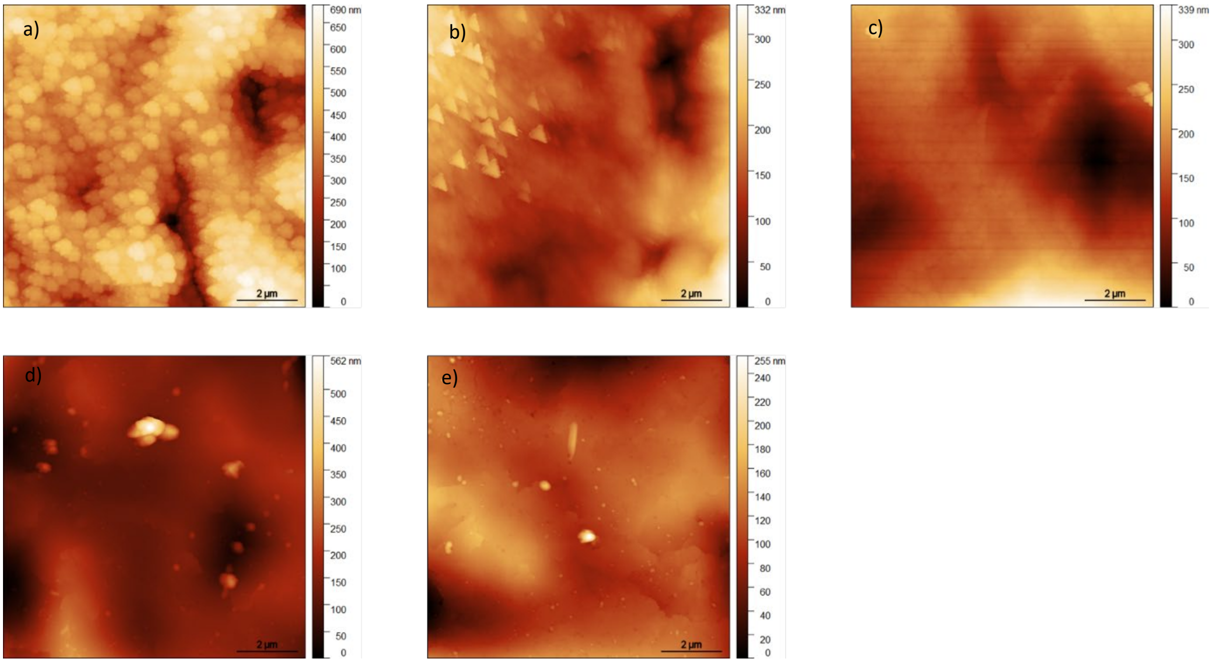

Figure S8. AFM images of a) pure PDMA n=5 film (RMS roughness 104.30 nm), b) PDMA film with 0.75MACl additive film (RMS roughness 49.63 nm), c) PDMA0.7PA0.6 film (RMS roughness 56.7 nm), d) PDMA0.7PA0.6_0.75MACl film (RMS roughness 49.93 nm), e) PDMA0.7PA0.6_0.75MACl+hot-precursor film (RMS roughness 27.86 nm). The scale bars are all 2 µm.

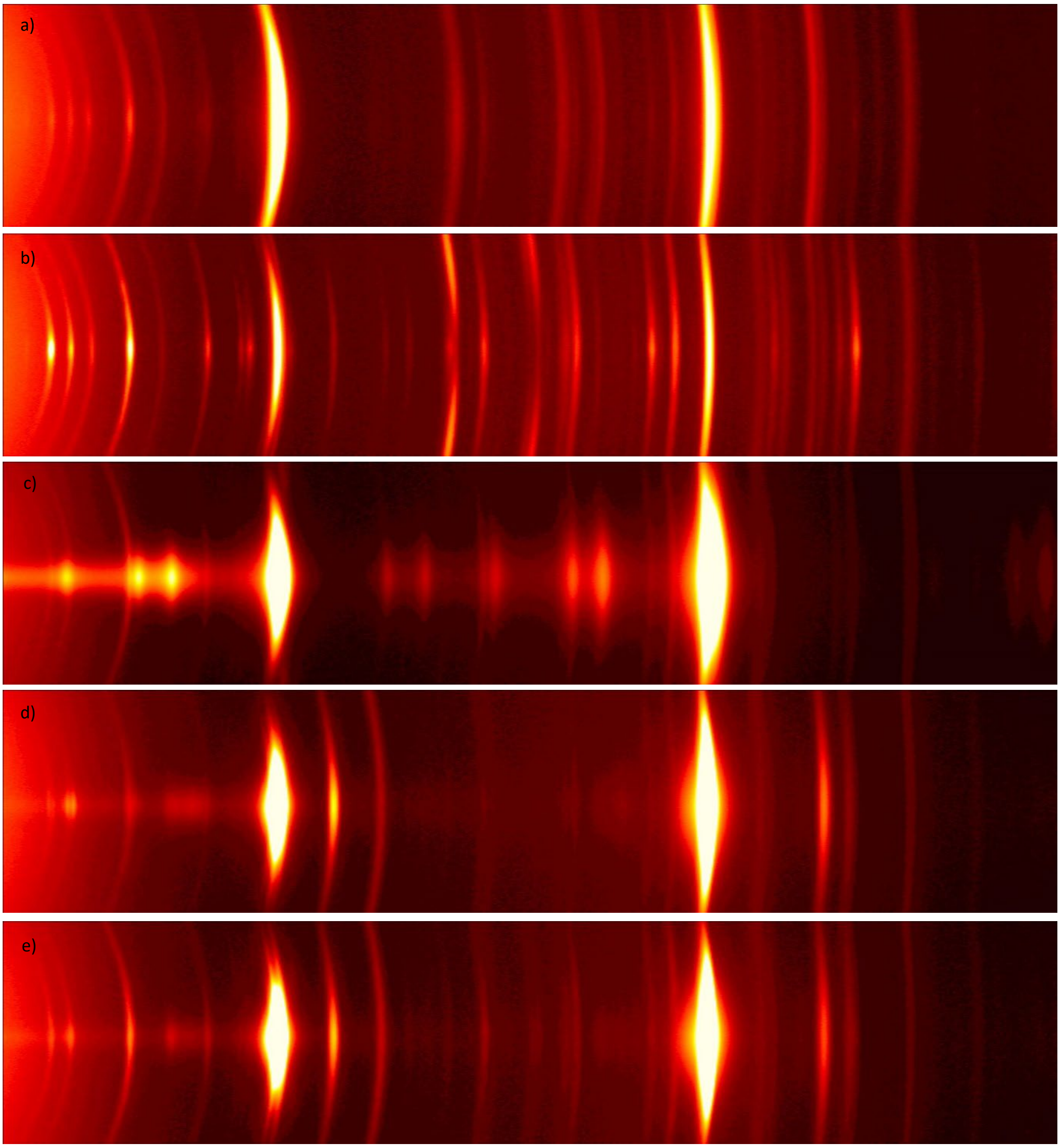

Figure S9. GIWAX images of a) pure PDMA n=5 film, b) PDMA film with 0.75MACl additive film, c) PDMA0.7PA0.6 film, d) PDMA0.7PA0.6_0.75MACl film, e) PDMA0.7PA0.6_0.75MACl+hot-precursor film.
